# Cardio-hepatic risk assessment by CMR in liver transplant candidates; advancing beyond a proof of concept

**DOI:** 10.1186/1532-429X-18-S1-P145

**Published:** 2016-01-27

**Authors:** Sahadev T Reddy, Ngoc L Thai, Jose Oliva, Kusum B Tom, Michael K Dishart, Mark Doyle, June A Yamrozik, Ronald Williams, Moneal Shah, Anil singh, Robert W Biederman

**Affiliations:** grid.413621.30000000404551168Cardiac MRI, Allegheny General Hospital, Pittsburgh, PA USA

## Background

The pre-operative workup of orthotopic liver transpantation (OLT) patents is practically complex given the need for separate cardiac, vascular, and abdominal imaging. Precise diagnosis and staging of hepatocellular carcinoma (HCC) is crucial in the selection and timing orthotopic liver transplantation (OLT) in cirrhotic pts. We have recently demonstrated in our 'Proof of Concept' study in determining the short term value of a 'One Stop Shop' approach combining the attributes of a nuclear stress, echocardiogram and abdominal MRI into one single, same day, pre-op CMR evaluation. However, this approach requires further validation, as detection of HCC as well as cardiovascular risk assessment is critically important in these pts. We hypothesize in this intermediate term and larger study that CAD assessment and HCC detectability is independent of whether the study is performed as part of a larger One Stop Shop CMR or as a traditional focused MRI.

## Methods

In this observational study, patients underwent SSFP sequences, stress CMR, thoracoabdominal MRA, and abdominal MRI on a standard MRI scanner on a single day while a matched cohort underwent standard separate MRI, Echocardiography and Nuclear stress testing over a 2-3 day protocol. CMR Pharmacological stress test was performed using vasodilators or dobutamine.

## Results

Over 6 years, 218 consecutive liver transplant candidates (mean age 58 ± 9 years, 40% female, mean MELD score:16 ± 8; range: 6-40) underwent 289 pre-op evaluations in the cardiac MRI suite. All referred pts completed standard dynamic CMR, 89% completed stress CMR, 89% completed LGE for viability, 88% completed liver MRI and 88% completed MRA. The mean duration of the entire study was 72∫ ± 5 min while 91%of pts were able to complete the entire exam. Among all 218 patients, 12 required follow-up coronary angiography due to abnormal CMR (4 patients had significant ischemia and required intervention, one had moderate AS). A total of six pts underwent RHC to evaluate pulmonary hypertension based on cardiac MRI findings and were found to have mild to moderate PH. A total of 38 (17%) pts proceeded to OLT. There were 53 (24%) ascertained mortalities in the non-transplant group and 6 deaths in the transplanted group; non from CAD. Explant pathology confirmed/exclusion of HCC in explanted livers; 98% detected on liver MRI but not in 1 pt who had HCC lesions that were ≤ 2 mm. No complications during CMR examination were encountered.

## Conclusions

Our study confirms that CMR evaluation of the liver along with cardiac evaluation is equivalent to traditional testing for the detection of HCC lesions as well as adding a pre-operative cardiac risk evaluation supplanting the need for separate Echocardiography and Nuclear stress testing. This is an important next step towards the long-term validation of the CMR One-Stop-Shop concept for pre-op evaluation for potential liver transplant patients improving patient throughput and satisfaction while eliminating radiation and reducing total costs.

